# Proviral ALV-LTR Sequence Is Essential for Continued Proliferation of the ALV-Transformed B Cell Line

**DOI:** 10.3390/ijms231911263

**Published:** 2022-09-24

**Authors:** Swagata Roy, Megha Sravani Bondada, Yaoyao Zhang, Katy Moffat, Venugopal Nair, Yongxiu Yao

**Affiliations:** 1 The Pirbright Institute, Pirbright, Woking GU24 0NF, UK; 2 The Jenner Institute Laboratories, University of Oxford, Oxford OX1 3SZ, UK; 3 Department of Zoology, University of Oxford, Oxford OX1 3SZ, UK

**Keywords:** avian leukosis virus, integration, LTR, *c-myc*

## Abstract

Avian leukosis virus (ALV) induces B-cell lymphomas and other malignancies in chickens through insertional activation of oncogenes, and *c-myc* activation has been commonly identified in ALV-induced tumors. Using ALV-transformed B-lymphoma-derived HP45 cell line, we applied in situ CRISPR-Cas9 editing of integrated proviral long terminal repeat (LTR) to examine the effects on gene expression and cell proliferation. Targeted deletion of LTR resulted in significant reduction in expression of a number of LTR-regulated genes including *c-myc*. LTR deletion also induced apoptosis of HP45 cells, affecting their proliferation, demonstrating the significance of LTR-mediated regulation of critical genes. Compared to the global effects on expression and functions of multiple genes in LTR-deleted cells, deletion of *c-myc* had a major effect on the HP45 cells proliferation with the phenotype similar to the LTR deletion, demonstrating the significance of *c-myc* expression in ALV-induced lymphomagenesis. Overall, our studies have not only shown the potential of targeted editing of the LTR for the global inhibition of retrovirus-induced transformation, but also have provided insights into the roles of LTR-regulated genes in ALV-induced neoplastic transformation.

## 1. Introduction

Avian leukosis viruses (ALV), belonging to different envelope subgroups of avian retroviruses, are major pathogens associated with different types of neoplastic diseases in poultry [1]. Similar to other retroviruses, ALV integrates into the host genome to induce tumorigenesis by modifying the expression of host genes. ALV long terminal repeat (LTR), with strong promoter-enhancer and other regulatory elements, drive the expression of genes adjacent to the insertion site through the process referred to as insertional activation, first demonstrated with the *c-myc* oncogene [2]. In addition to the ALV-LTR-induced enhanced expression of the *c-myc*, insertional activation of other genes such as *c-bic* resulting in synergistic enhancement of lymphomas, particularly in the late stages of tumor development, has also been reported [3]. Although the functional nature of the *c-bic* locus was not clear at the time of its discovery, subsequent studies have identified the multifunctional *miR-155* as the gene product [4,5], and detailed studies highlighting the direct role of *miR-155* in many cancers have recently been reviewed [6,7]. Retroviral integrations near host genes can result in deregulation of gene expression through different pathways, including enhancer or promoter insertion or mRNA 3’ end substitution, as well as through insertional disruption [8]. ALV-mediated induction of lymphoma through *c-myc* occurs mainly by direct transcriptional activation as well as through transduction of the *v-myc* oncogene by acutely transforming viruses such as MC29 [9]. Members of the Myc family, which include *c-myc*, *L-myc,* and *N-myc*, are powerful transcription factors that regulate cellular growth and proliferation [10]. This family of oncogenes is also actively associated with the development of multiple cancers [11,12]. The direct role of *c-myc* as an oncogene in the induction of cancer was first demonstrated by the insertional activation of the *c-myc* promoter in ALV-induced B-cell lymphomas [2] and subsequently confirmed in other tumors [13]. Myc transcriptional regulator function occurs through the specific interaction with the palindromic DNA sequence (CACGTG), called ‘E-Box’, with the help of myc-associated factor X (MAX) as a heterodimer [10]. 

While *c-myc* is indeed one of the most common integration sites in ALV-induced tumors [13], several other integration sites in genes such as *CTDSPL*, *CTDSPL2*, *MYB*, *TERT*, *MET*, *EGFR*, *TNFRSF1A*, *MEF2C*, *CTDSPL*, *TAB2*, *RUNX1*, *MLL5*, *CXorf57*, and *BACH2* have been also identified in ALV-induced B-cell lymphomas [14,15,16,17]. Comprehensive identification of the integration sites in ALV-transformed cells is important in understanding the global changes in gene expression directly associated with retroviral integration. Several methods have been used for the identification of retroviral integration sites in the genome [18], including inverse PCR [19], splinkerette PCR [20] and Cas9 enrichment [21] together with the next generation sequencing (NGS) for high throughput identification of integration sites. In order to examine the direct effect of the ALV-LTR-induced gene regulation on the transformation phenotype of the HP45 cell line, we carried out CRISPR-Cas9-mediated targeted knockdown (KD) of a region of the ALV-LTR. Our studies showed that targeted editing of the ALV-LTR had a dramatic effect on the proliferation of the HP45 cell line as well as the dysregulation of the expression of LTR-regulated genes, demonstrating the broad role of the integrated proviral ALV-LTR on the transformed phenotype of the HP45 cell line. Targeted editing of *c-myc* showed that *c-myc* is a major contributor of the observed phenotypical changes induced by the ALV-LTR deletion. 

## 2. Results

### 2.1. ALV-LTR Editing Downregulates Expression of Insertionally Activated Genes 

Using an ALV-transformed B-cell line with the proviral integration in the *c-myc* locus, we first examined the *c-myc* expression in the HP45 cell line by RT-qPCR with myc-specific primers (Table 1). Primary B cells (isolated from the chicken bursa of Fabricius) expressing physiological levels of *c-myc* were used as a control. Analysis of the RT-qPCR data, relative to the *GAPDH* [22], showed that *c-myc* expression was almost 28-fold higher in the HP45 cells compared to the primary B cells (Supplementary Figure S1A). This suggested that insertional activation of *c-myc* through the LTR of the integrated ALV provirus contributes to the enhanced *c-myc* gene expression in the transformed HP45 cell line. 

After demonstrating the enhanced expression of *c-myc* in the HP45 cells, we investigated whether insertional activation through the strong LTR promoter was driving the overexpression of *c-myc* in these cells. We applied targeted CRISPR/Cas9 editing of the integrated proviral LTR using the gRNAs listed in Table 2. The efficiency of the LTR editing was analyzed by PCR, using specific forward and reverse primers (Table 1) on genomic DNA from the edited HP45 cell population at different time points post transfection. A reduction in the size of the amplified DNA, compared to that seen in the non-targeting SgA control (SgA-NT), demonstrated the targeted editing of the LTR (Supplementary Figure S1B, top panel). Sequence analysis of the purified PCR products further confirmed the deletion of the target region compared with that of wild-type LTR sequence (Supplementary Figure S1C).

**Figure 1:**
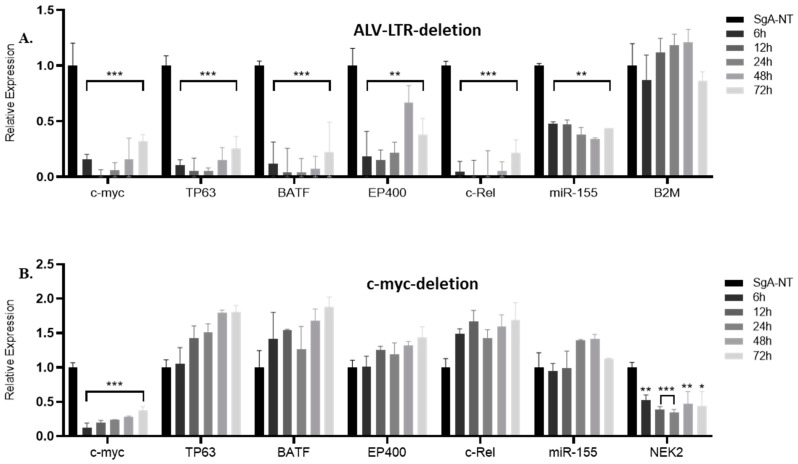
Expression of ALV-LTR-activated genes following ALV-LTR or *c-myc* deletion in HP45. RT-qPCR analysis of *c-myc*, *TP63*, *BATF*, *EP400*, *c-Rel* gene and *miR-155* expression in ALV-LTR deleted (**A**) or *c-myc* deleted (**B**) cells and the control cells. Cas9 expressing HP45 cells were transfected with non-targeting control gRNA SgA-NT or ALV-LTR-gRNAs/c-myc-gRNAs. For the ALV-LTR-gRNAs/c-myc-gRNAs transfections, the transfected cells were harvested at 6, 12, 24, 48, and 72 h post transfection, and RNA was extracted, followed by RT-qPCR analysis using gene-specific primers and SYBR green reagents. Relative expression of miR-155 was measured using miR155-specific TaqMan probe and primers (Applied Bioscience). The data were plotted as 2^−^^ΔΔ^^CT^ where transcript levels were normalized against *GAPDH* for *c-myc*, *TP63*, *BATF*, *EP400*, and *c-Rel* and against U6-small nuclear RNA (U6-SnRNA) for *miR-155*. The value corresponding to the level of each gene/miRNA in SgA-NT transfected cells harvested at 72 h post transfection was set as 1. All data are representative of three independent experiments and the statistical significance was measured by *t*-test using GraphPad software, from 3 independent experiments (* *p* < 0.05; ** *p* < 0.01; *** *p* < 0.001).

As one of the most important genes regulated by insertional activation, we first examined the effect of LTR editing on the expression of *c-myc* by RT-qPCR. The data were plotted as 2^−ΔΔCT^, and the gene expression was compared with the expression levels relative to SgA-NT guide-RNA, using *GAPDH* as an internal control. We observed a highly significant reduction in *c-myc* expression in LTR-edited cells at different time points (Figure 1A). We also examined the expression of other LTR-regulated genes identified by Targeted locus amplification (TLA) analysis or integration junction PCR (manuscript in preparation). Targeted editing of LTR in HP45 cells resulted in downregulation of LTR-regulated genes such as *BATF* (basic leucine zipper ATF-like transcription factor), *TP63* (Tumor protein 63), *EP400* (E1A Binding protein P400), *c-Rel,* and *miR-155* expressed from the *c-Bic*. However, the expression of B2M (Beta-2-microglobulin), one of the stably expressed reference genes [23] and not targeted by ALV-LTR, has not been affected (Figure 1A).

### 2.2. Knockdown of c-myc Did Not Affect Expression of Other LTR-Regulated Genes 

Editing of the LTR had a downward effect on the expression of multiple genes regulated by LTR. To confirm that the changes in gene expression were not an indirect effect of the reduced expression of key LTR-regulated genes, such as *c-myc*, we carried out targeted editing of *c-myc* using specific gRNAs (Table 2, Supplementary Figure S1B, bottom panel and S1–D). The expression levels of different LTR-regulated genes were then measured by RT-qPCR using SYBR green master mix and gene-specific primers (Table 1). Targeted editing of the *c-myc* resulted in a significant reduction in *c-myc* expression at different time points (Figure 1B). However, editing of *c-myc* did not affect the expression of any of the other LTR-regulated genes, including *miR-155* (Figure 1B), confirming that the changes in LTR-regulated gene expression were indeed a direct effect of LTR deletion and not *c-myc* downregulation. Interestingly, the expression of the NEK2 gene, one of the *c-myc* target genes identified by the *c-myc* ChIP-Seq assay of HP45 (manuscript in preparation), is downregulated after *c-myc* deletion, further confirming that the direct effect of LTR deletion on its integrated genes is not due to *c-myc* downregulation. 

### 2.3. LTR Editing Inhibits Proliferation of HP45 Cells 

**Figure 2:**
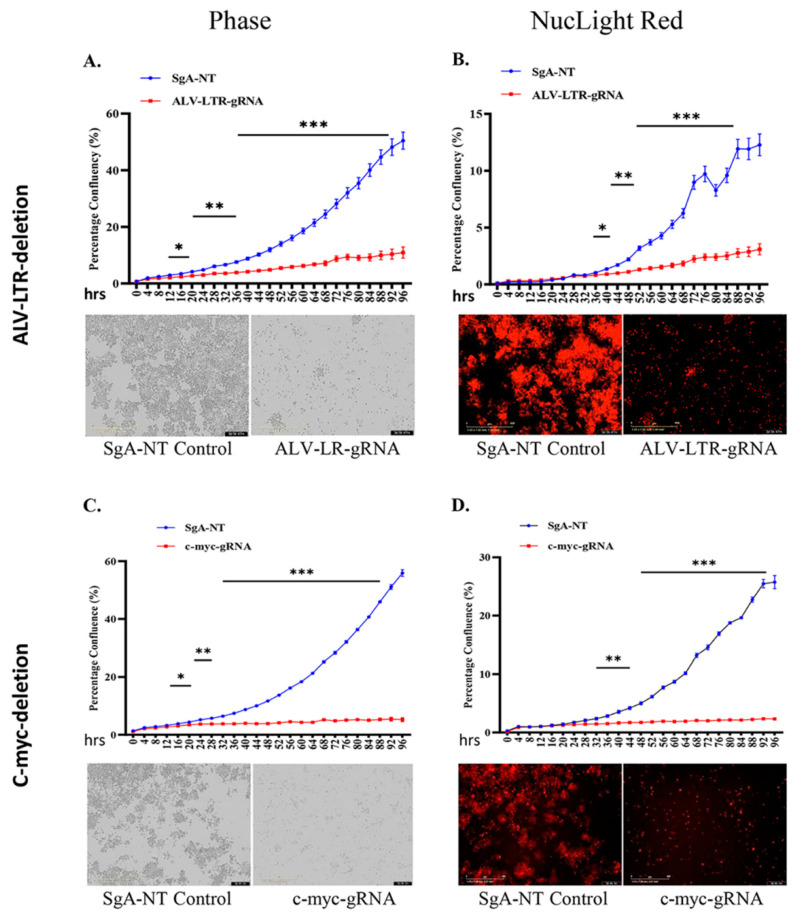
Proliferation of gRNA-transfected cells monitored in real time using the IncuCyte S3 live imaging system. gRNAs targeting ALV-LTR (**A**,**B**) or *c-myc* (**C**,**D**) transfected cells were subjected to proliferation assay using NucLight Rapid Red Reagent in IncuCyte S3 imaging system. Phase (**A**,**C**) and red (**B**,**D**) object confluency of each cell population were determined every 4 h for 96 h from five separate regions per well and four wells per sample by IncuCyte S3 and compared with SgA-NT control. Growth curves (upper panels) are shown as mean ± standard error (SE) representative of three independent experiments. Asterisk (*) indicates statistically significant differences between gRNA-edited cells and SgA-NT cells at different time points. * *p* <0.05; ** *p* < 0.01; *** *p* < 0.001. Phase and red object images (bottom panels) are representative of 72 h time points. The scale bar, 400 μm.

During LTR editing of HP45 cells, we observed that LTR-gRNA electroporated cells did not increase in numbers when compared to the SgA-NT electroporated control cells. This suggested that LTR editing also impacted cell proliferation. To examine this further, edited cell populations were subjected to a proliferation assay using IncuCyte^®^ NucLight Rapid Red Reagent. Dynamic changes in the proliferation of HP45-Cas9 cells electroporated with LTR-gRNAs, or SgA-NT control gRNA, were continuously monitored in IncuCyte S3 and analyzed using IncuCyte S3 2018C software. Data plotted as percentages of phase object confluency (Figure 2A upper panel) and red object confluence (Figure 2B upper panel) confirmed the reduced proliferation of the LTR-edited cells from the early time points, with highly significant differences (*p* < 0.001) observed at later time points. The flat growth curve of the LTR-edited cell population, compared to the rapidly proliferating control cells, was also reflected in the images taken at 72 h time point for phase (Figure 2A, lower panel) and red fluorescence (Figure 2B, lower panel) channel in the IncuCyte S3.

### 2.4. Downregulated c-myc Contributes to Proliferation Defect of LTR-Edited HP45 

Having demonstrated the proliferation defect of the LTR-edited HP45 cells, we wanted to identify the LTR-regulated gene(s) that directly contributed to this phenotype. In particular, we wanted to examine whether the reduced expression of *c-myc* (Figure 1A) in the LTR-deleted cells contributed to the inhibition of proliferation. For this, we used the IncuCyte S3 to measure the dynamic changes in the proliferation of c-myc-knockout HP45 cells. We observed a significant defect in the proliferation of *c-myc* deleted HP45 cells (Figure 2C,D, phase and NucLight rapid red, respectively) in a pattern very similar to LTR-deleted cells. Control cells transfected with SgA-NT showed no defect in proliferation. This indicates that *c-myc* is a key contributor to proliferation defects in HP45 with LTR deletion.

### 2.5. Knockdown of LTR and c-myc Induces Apoptosis in HP45 Cells 

**Figure 3:**
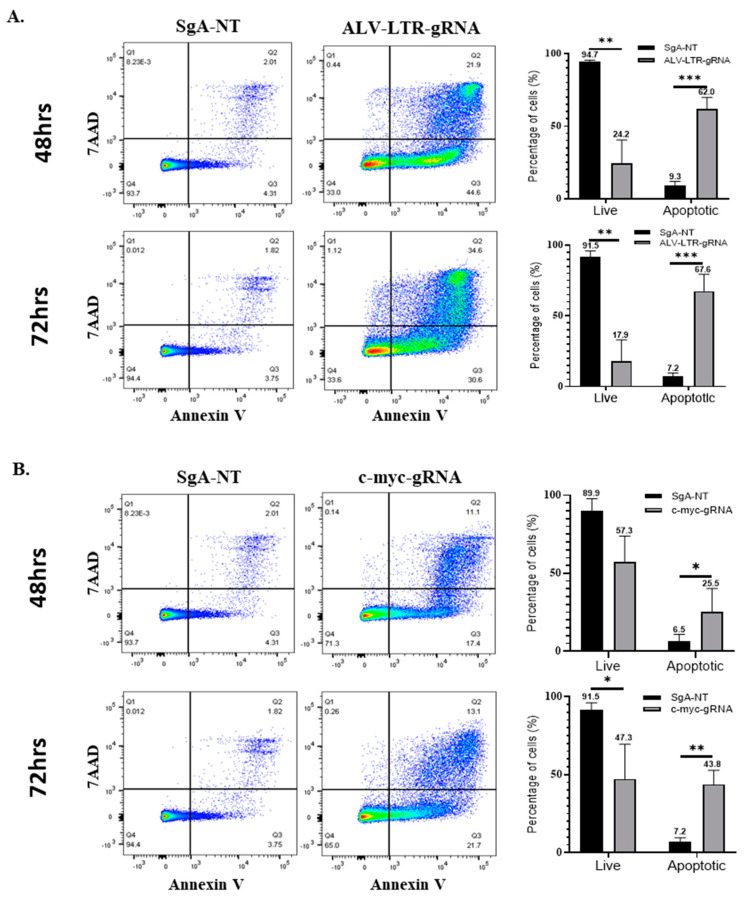
Apoptosis Assay and viability assay of gRNA-transfected cells. SgA-NT (left panels) or gRNAs targeting ALV-LTR (**A**) and c-myc (**B**) (middle panels) transfected cells were stained with Annexin-V-FITC and 7AAD at 48 h (upper panels) and 72 h (lower panels) post transfection and analyzed in FlowjoX with data collected on a BD Bioscience LSR Fortessa. The histogram (right panels) shows the percentage of live and apoptotic cells calculated from the flow cytometry data. Each experiment has been performed a minimum of three times, multiple *t*-tests have been performed, and the statistical significance was calculated using the Holm-Sidak method. Asterisk (*) indicates statistically significant differences between gRNA-edited cells and SgA-NT cells at different time points. * *p* <0.05; ** *p* < 0.01; *** *p* < 0.001.

Having demonstrated a significant defect in the proliferation rates of HP45 cells with deletions in the LTR and *c-myc*, we explored the possible mechanisms. During the early stage of the experiments, we observed that the gene-deleted cell populations were not only defective in proliferation but also dying in significant numbers, especially in the case of ALV-LTR-edited cells. In order to quantify the percentage of dying cells in the knockout populations, these cells were analyzed for apoptosis using FITC Annexin-V Apoptosis Detection Kit with 7-AAD. HP45 cells, harvested at 48 h and 72 h post electroporation with LTR- or c-myc-specific gRNAs or SgA-NT gRNA, were stained according to the manufacturer’s protocol, and data were collected using DIVA 8 software and a BD biosciences LSR Fortessa. ALV-LTR-treated cells induced a marked increase in early and late apoptosis compared to control cells. The percentage of cells that were apoptotic at 48 and 72 h post electroporation with LTR-specific gRNAs was 62% (Figure 3A, top panel) and 67.6% (Figure 3A, bottom panel), respectively. In comparison, control cells showed only 9.3% and 7.2% apoptotic cells at the same time points. These results showed that targeting ALV-LTR not only slows down the proliferation rate but also increases the apoptosis of the cell population. Targeted editing of *c-myc* also induced apoptosis, but at a lower rate when compared to LTR-deleted cells. The percentage of apoptotic cells detected in *c-myc* knockdown were 25.5% and 43.8%, compared to control cells with 6.5% and 7.2% apoptotic cells at 48 and 72 h, respectively (Figure 3B).

## 3. Discussion

It has been reported that there is a strong association between retroviral integration, oncogenic activation, and tumorigenesis, not only in humans but in other species as well. Integration of ALV into various cellular proto-oncogenes such as *c-myc*, *MYB*, *TERT*, and *RUNX1* triggers the induction of tumors causing lymphoid, myeloid, or erythroid tumors, depending on the cell types affected [1,13,16]. The most common site of ALV integration in bursal lymphomas and derivative cell lines corresponds to the 5’ flanking region of the *c-myc* gene, with the proviral integration occurring mainly within the 5 kb region of the transcriptional start site [24]. In many of these lymphomas, there is also second integration in the *c-bic* oncogene that encodes the oncogenic microRNA miR-155 [4,25,26,27,28,29].

In this study, we have used CRISPR-Cas9-based targeted editing of the integrated ALV proviral genome to demonstrate the broad effect of the LTR on the expression of ALV-LTR integration-associated genes. We have examined c-*myc, TP63, BATF, EP400, c-REL,* and *miR155* gene expression levels in HP45 cells where the LTR region was targeted using specific guide-RNAs. Among these genes, the roles of *c-myc* and *TP63* on cell proliferation or growth have been published [30,31]. In this study, we investigated the effect of knockdown of ALV-LTR and c-*myc* on HP45 cell proliferation using the IncuCyte S3 live cell analysis system. These studies showed significant proliferation defects in both ALV-LTR and *c-myc* KD cells compared to control cells, indicating that *c-myc* is a major contributor to the observed proliferation defect after the ALV-LTR deletion. ALV-LTR KD cells exhibited high levels of cell death when viewed under the microscope. Although *c-myc* has a prominent role in driving apoptosis [32], it has also been reported to mediate the inhibition of apoptosis [33]. Our data have shown that KD of *c-myc* also induced apoptosis of HP45 cells. Interestingly, the apoptosis levels were significantly higher when targeting the LTR region compared to the cells where *c-myc* was targeted. This suggests that in addition to *c-myc*, other LTR-activated genes, such as *TP63,* may also contribute to the apoptosis induced by ALV-LTR editing. 

The oncogene *c-myc* has been implicated in driving neoplastic transformation by other oncogenic viruses. Epstein-Barr Virus (EBV) is highly pathogenic and causes a number of B-cell malignancies. It has been reported that a high level of *c-myc* expression reduces the latent membrane protein 1 (*LMP1*) expression, which is essential for the EBV infected B-cell immortalization; thus, the effect of EBV infection in primary B cells in the first few weeks is not evident [34]. Recent data has also demonstrated that *c-myc* controls the EBV lytic switch by remodeling the viral genome [35]. 

In summary, we have demonstrated the successful application of CRISPR/Cas9-based targeted in situ editing of the LTR of the integrated retroviral genome as well as the insertionally activated major oncogene *c-myc* in an avian B-cell lymphoma-derived tumor cell line. Overall, our data suggest that KD of ALV-LTR has a broad effect on the ALV integration-related gene expression, proliferation, and apoptosis. KD of *c-myc* also had a similar functional effect indicating its importance among the multiple insertionally activated target genes. The lower level of apoptosis induced by *c-myc* deletion compared to ALV-LTR deletion suggested that other ALV-LTR insertionally activated genes may also contribute to the overall apoptosis effect with ALV-LTR deletion. The exact role and degree of contribution by other integrated genes need further investigation. While the targeted editing of genes in transformed cell lines in situ can provide insights into the gene functions and downstream regulatory pathways, these approaches could also be utilized in developing a therapeutic target by directly aiming to inactivate the LTR region and indirectly targeting many genes, thus reducing the proliferation and growth of the cancerous tissues. 

## 4. Materials and Methods

### 4.1. Cell Culture and Generation of HP45-Cas9 Cell Line 

ALV-transformed HP45 cells [36] were cultured in RPMI 1640 (Sigma-Aldrich, Burlington, MA, USA) supplemented with 10% tryptose phosphate broth (TPB), 10% heat inactivated FBS (Sigma-Aldrich, Burlington, MA, USA), 10 mM Sodium pyruvate (Sigma-Aldrich, Burlington, MA, USA), 50 mM 2-mercaptoethanol (GIBCO, Waltham, MA, USA) and 100 units/mL penicillin and streptomycin (GIBCO, Waltham, MA, USA), kept at 38.5 °C incubator with 5% CO_2_ and humidity. 

To generate the stable HP45 cell line expressing Cas9, 1 × 10^6^ of HP45 cells were resuspended in 96 µL of Opti-MEM medium (GIBCO, Waltham, MA, USA) and electroporated with 10 µg of pX459-V2.0 plasmid (Addgene, Watertown, MA, USA) using a NEPA21 electroporator (Sonidel Ltd., Dublin, Ireland). After selection with 1 µg/mL puromycin (Sigma-Aldrich, Burlington, MA, USA) for 7 days, single cell clones were isolated using fluorescence-activated cell sorting using FACSAria IIu (BD Bioscience, Wokingham, Berkshire, UK). The single cell clone with Cas9 expression detected by Western blotting with anti-Flag antibody (Supplementary Figure S2) was grown and stored in liquid nitrogen for further studies.

### 4.2. Guide-RNA Design

The gRNAs targeting ALV-LTR and *c-myc* were designed using http://crispor.tefor.net/, accessed on 8 March 2019, and the MIT specificity score was considered not less than 95%. A two-part guide-RNA system containing crRNA and tracrRNA guide complex was used for gene editing. Sequences of ALV-LTR [37] and *c-myc* [38] from HP45 genomic DNA were confirmed by sequencing of the PCR products. The gRNAs designed to target ALV-LTR and *c-myc* (Table 2) were produced by Integrated DNA Technologies (IDT, Diego, CA, USA). Aliquots of lyophilized crRNA and tracrRNA pellets were resuspended in duplex buffer (IDT) at 200 µM concentration and stored at −80 °C.

### 4.3. Genomic DNA Isolation

Edited HP45-Cas9 cells and SgA-NT transfected control cells were harvested after electroporation at different time points. Cell pellets were washed twice with PBS and resuspended in 100 µL of 1× Proteinase K-based DNA extraction buffer (10 mM Tris-HCL, 1 mM EDTA, 25 mM NaCl, and 200 µg/mL Proteinase K) and incubated at 65 ℃ for 30 min followed by proteinase K inactivation at 95 ℃ for 2 min and stored at −20 ℃. A total of 1 µL of genomic DNA was used for PCR.

### 4.4. Electroporation of gRNAs into HP45-Cas9 Cell Line for Targeted Editing

To knockout the ALV-LTR region or *c-myc* gene, 2 × 10^6^ of HP45-Cas9 cells were resuspended in 96 µL Opti-MEM medium. ALV-LTR-gRNAs 1-3 or c-myc-gRNAs 1-2 were mixed with equimolar of tracrRNA to reach a final duplex concentration of 100 µM. The mix was incubated at 95 °C for 5 min and allowed to cool to room temperature until use. The duplex was mixed with the cell suspension in a total volume of 100 µL and electroporated using a NEPA21 electroporator (Nepa Gene Co., Ltd., Ichikawa, Japan). Electroporated cells were immediately transferred into a 6-well plate containing 2 mL of complete growth media. Cells were harvested at 6 h, 12 h, 24 h, 48 h, and 72 h intervals for RT-qPCR and PCR analysis. The proliferation assay was set up immediately following the electroporation process. The cells were harvested at 48 h and 72 h time points and analyzed in an apoptosis assay.

### 4.5. RNA Extraction, cDNA Synthesis, and RT-qPCR 

Total RNA was extracted using TRIzol Reagent (Life Technologies, Paisley, UK) following the manufacturer’s protocol. The RNA was quantified using a Nanodrop (ThermoFisher Scientific, Gloucester, UK) and diluted to 500 ng/µL with RNase-free water for cDNA synthesis. c-DNAs were synthesized using High-Capacity RNA-to-cDNA Kit (Applied Biosystem^TM,^ Waltham, MA, USA), and 500 ng RNA was used for each reaction. qPCR was performed using Luna^®®^ Universal qPCR Master Mix (New England Biolabs, Herts, UK), which uses real-time fluorescence of a double-stranded DNA (dsDNA) binding dye, commonly SYBR Green, and 10 µM forward and reverse primer specific to each gene (Table 1). The transcripts were normalized to endogenous GAPDH (glyceraldehyde-3-phosphate dehydrogenase) and to the experimental SgA-NT control and plotted as 2^−ΔΔCt^ [22].

To determine the microRNA (miRNA) expression level, total RNA was extracted using a miRNeasy Mini Kit (Qiagen, Hilden, North Rhine-Westphalia, Germany), following the manufacturer’s protocol. The RNA was quantified using a Nanodrop (Thermo Fisher Scientific, Paisley, Scotland, UK) and diluted to 10 ng/µL with RNase-free water for cDNA synthesis. cDNAs were synthesized using a TaqMan™ MicroRNA Reverse Transcription Kit (Applied BiosystemTM, Waltham, MA, USA). TaqMan™ MicroRNA Assay RT-hsa-miR-155 and RT-U6 snRNA primers were used with 50 ng of miRNA samples for each reaction. For qPCR, ABsolute Blue qPCR Low ROX master mix was used along with TaqMan TM-hsa-miR-155 and TM-U6 snRNA probe. For the relative quantification of miRNA155, Ct values were normalized to the expression level of endogenous U6-SnRNA and the experimental SgA-NT control and plotted as 2^−^^ΔΔCT^. The value corresponding to the level of miR-155 in SgA-NT transfected HP45-Cas9 was set as 1 for calibration.

### 4.6. Proliferation Assay with IncuCyte NucLight Rapid Red Reagent 

After electroporation of HP45-Cas9 cells with gRNAs targeting different sequences and SgA-NT gRNAs, 5000 cells/well were plated for each sample into a 96-well plate in quadruplicates and measured for proliferation with IncuCyte NucLight Rapid Red reagent (1:500 dilution) in an IncuCyte (IncuCyte S3 Sartorius, ESSEN BioScience, Hertfordshire, UK). The plate was scanned from five separate regions per well using a 10× objective every 4 h. The phase object confluence and the red object count data were plotted using IncuCyteS3-2018C software. The IncuCyte data were analyzed by two-way analysis of variance (ANOVA) with Tukey’s multiple-comparison test using GraphPad Prism version 8 Software. The results are shown as means ± standard errors (SE) of results from four replicates, each with five separate regions per well and representative of three independent experiments. *p* values of <0.05 were considered to be significant.

### 4.7. B-Cell Isolation from Bursa of Fabricius (BF) 

Freshly collected BF tissues were washed 2–4 times with PBS before digesting in 5 mL (8 mg/mL) collagenase D (Sigma-Aldrich, Burlington, MA, USA) in 1× Hanks Balanced Salt Solution (HBSS) with calcium (Thermo Fisher Scientific, Paisley, Scotland, UK) at 37 ℃ for 15–20 min. Digested tissues were passed through a 100 µM cell strainer and centrifuged at 1100 rpm for 10 min, and the pellet was resuspended in 10 mL of complete IMDM media (with 2% chicken serum, 8% FBS, β-mercaptoethanol, streptomycin, and insulin transfer). Cells were carefully layered on 5 mL Histopaque (Sigma-Aldrich, Burlington, MA, USA) and centrifuged at 2000 rpm for 20 min at 4 ℃. Cells from the interface were collected and resuspended in cold PBS followed by centrifuge at 1800 rpm for 10 min at 4 ℃. The cells were washed twice the same way and resuspended in an appropriate volume of complete IMDM media. 

### 4.8. Apoptosis Assay 

Populations of ALV-LTR and c-myc-edited and SgA-NT control cells were subjected to Annexin-V apoptosis assay using FITC Annexin-V Apoptosis Detection Kit with 7-AAD (BioLegend, London, UK), as per manufacturer’s protocols. Briefly, cells washed twice with cold buffer (BioLegend, London, UK) were resuspended in Annexin-V binding buffer (0.25–1.0 × 10^7^ cells/mL), and 100 µL of cell suspension was mixed with 5 µL of FITC Annexin-V, followed by 5 µL of 7-AAD viability staining solution. After 15 min incubation at room temperature in the dark, Annexin-V binding buffer (400 µL) was added and analyzed by flow cytometry. Cells were identified, and data were collected using DIVA 8 software and a BD-LSR-Fortessa (BD bioscience, Wokingham, Berkshire, UK) flow cytometer, and 50,000 events were acquired for each sample. Data were analyzed using FlowJo v10.8 software (BD bioscience, Wokingham, Berkshire, UK). In brief, samples were gated on cells (SSC-A vs. FSC-A), singlets (SSC-A vs. SSC-H), and then early apoptotic cells were determined as AnnexinV-FITC-positive (blue 530/30 BP), and 7AAD-negative (YG 630/30 BP) and late apoptotic cells were determined as AnnexinV-FITC-positive and 7AAD-positive. Staurosporine (Abcam, Cambridge, UK)-treated cells were used as a positive control for early and late apoptosis.

## Figures and Tables

**Table 1:** Primers used in PCR and RT-qPCR.

Primer	Sequence (5′–3′)
c-myc-F	CTCCCCAGCAAGAACTACGAT
c-myc-R	GCAGATGAAGCTCTGGTTGAC
ALV-LTR-F	GGGGTAGGTGGCTATGATCG
ALV-LTR-R	CCCGAATAAGCGAGACGGAT
TP63-F	GATTGCACCTCCTAGCCACCTGATC
TP63-R	TGATGAGAATTGGGCGACGGTTCAT
BATF-F	TTGGAGAGCGAAGACCTGGAGAGAC
BATF-R	CAAGTTGGTTCTTAGCCGCCCCAG
c-Rel-F	CTGAACGTCGAGTCCTGTCTTTTCA
c-Rel-R	TCCACAGTTCTTATTCACACGGCAA
EP400-F	AGGAGTTAGTTGCTGTTGTGGATCA
EP400-R	TGTATGCATCCTCCCGAGTGTAGGT
B2M-F	AAGGAGCCGCAGGTCTAC
B2M-R	CTTGCTCTTTGCCGTCATAC
NEK2-F	TTATGTGCTCTCACGCCTCC
NEK2-R	TCCTGATCTCCGGCCTCTTT

F: forward; R: reverse.

**Table 2:** ALV-LTR and c-Myc guide-RNA sequence.

	Guide-RNA	Sequence (5′–3′)	Specificity
**ALV-LTR-gRNAs**	gRNA-1 (F)	CAGACGGGTCTAACACGGAT	99%
	gRNA-2 (R)	GGCGTTTATTGTATCGAGCT	99%
	gRNA-3 (F)	GTTGATTCCCTGACGACTAC	97%
**c-myc-gRNAs**	gRNA-1 (F)	CTACGATTACGACTACGACT	99%
	gRNA-2 (R)	CTTCCAGATGTCCTCGGACG	96%

F: forward; R: reverse.

## Supplementary Materials

The supporting information can be downloaded at: https://www.mdpi.com/article/10.3390/ijms231911263/s1.
