# Supplementary material for: Proviral ALV-LTR Sequence Is Essential for Continued Proliferation of the ALV-Transformed B Cell Line
Source: Int J Mol Sci. 2022 Sep 24;23(19):11263. doi: 10.3390/ijms231911263 (PMC9569804; doi:10.3390/ijms231911263)
Supplement: Supplementary file 1 [file ijms-23-11263-s001.zip › Supplementary Figure legend.pdf]

Supplementary Figure S1. Myc expression and deletion of ALV-LTR and c-myc by CRISPR/Cas9 editing in HP45 cells. A) c-myc gene expression was measured in primary B-cells, derived from chicken bursa and ALV transformed B-cell line HP45. Total RNA was extracted followed by cDNA synthesis and qPCR reaction using Myc primers and SYBR green reagent. The data was plotted as 2-ΔΔCT where transcript levels were normalized against GAPDH and primary B-cell as control. B) PCR amplification of the edited region, using ALV-LTR or c-myc specific primers located at the flanking region of the gRNA targeting sites using genomic DNA isolated from ALV-LTR-gRNAs, c-myc-gRNAs and SgA-NT transfected Cas9 expressing HP45 cells harvested at 6, 12, 24, 48 and 72 hrs post transfection as templates. The samples were run in a 2% agarose gel. C) Sequencing chromatograms of PCR products of LTR deleted (HP45-ΔLTR) and SgA-NT control bands (HP45-SgA). The junction sequence after cleavage and re-joining in HP45-ΔLTR and the corresponding sequence in the SgA-NT transfected HP45 are outlined in red. The sequence present between the outlined sequences in the SgA-NT transfected HP45 indicated the deleted region by CRISPR/Cas9. D) Sequencing chromatograms of PCR products of c-myc deleted (HP45-ΔMyc) and SgA-NT control bands (HP45-SgA). The junction sequence after cleavage and re-joining in HP45-ΔMyc and the corresponding sequence in the SgA-NT transfected HP45 are outlined in red. The sequence present between the outlined sequences in the SgA-NT transfected HP45 indicated the deleted region by CRISPR/Cas9.

Supplementary Figure S2. Detection of Cas9 expression on single clone of HP45 cell line stably expressing Cas9 by western blotting. Cell lysates of HP45-Cas9 along with HP45 were separated by SDS-PAGE, Western blotted, and probed with anti-Flag antibody, with α-tubulin as the loading control.
