# Supplementary figures and images for: Proviral ALV-LTR Sequence Is Essential for Continued Proliferation of the ALV-Transformed B Cell Line
Source: Int J Mol Sci. 2022 Sep 24;23(19):11263. doi: 10.3390/ijms231911263 (PMC9569804; doi:10.3390/ijms231911263)

## Slide 1
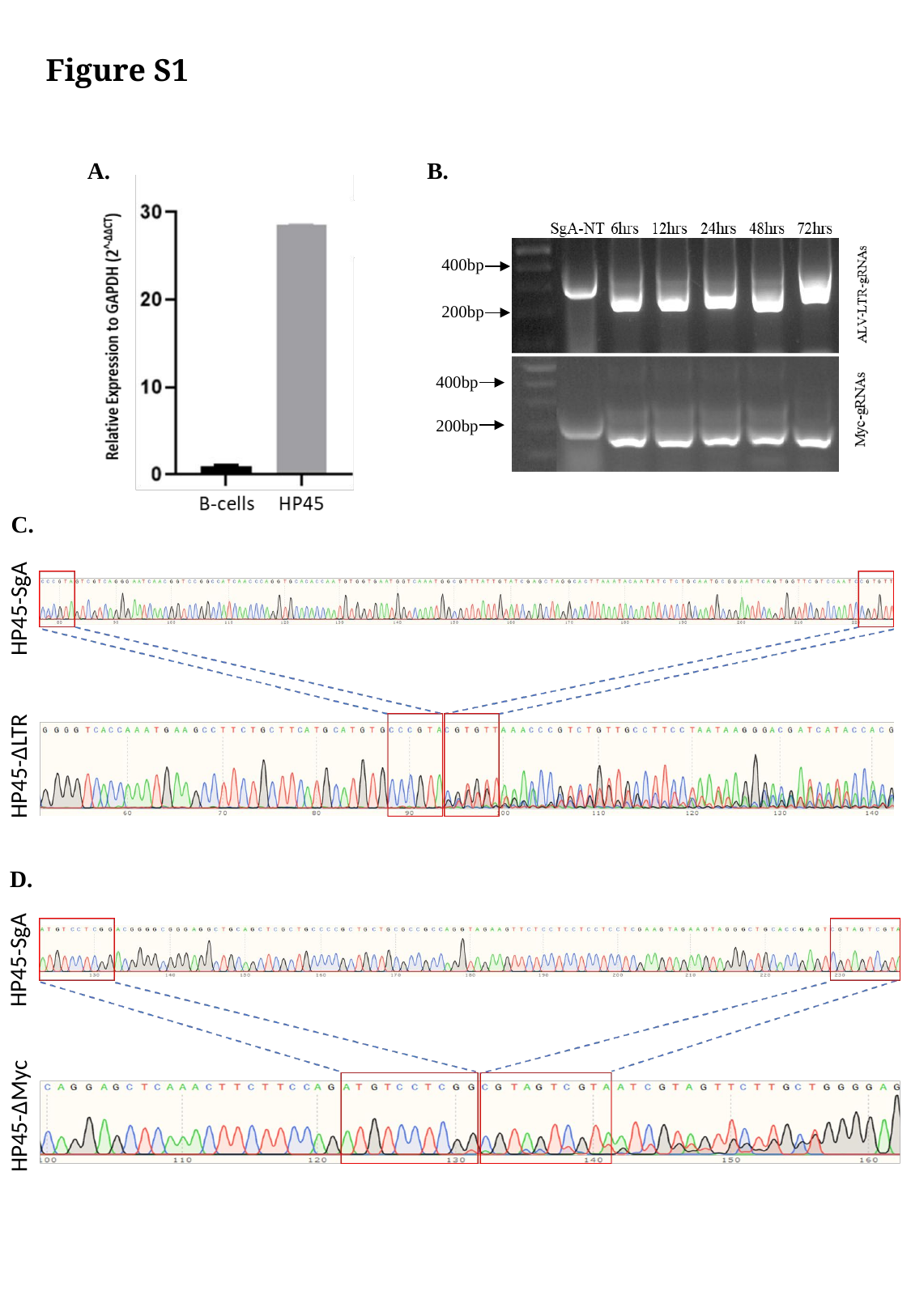

Figure S1
A.
B.
400bp
200bp
400bp
200bp
C.
HP45-SgA
HP45-ΔLTR
D.
HP45-SgA
HP45-ΔMyc

## Slide 2
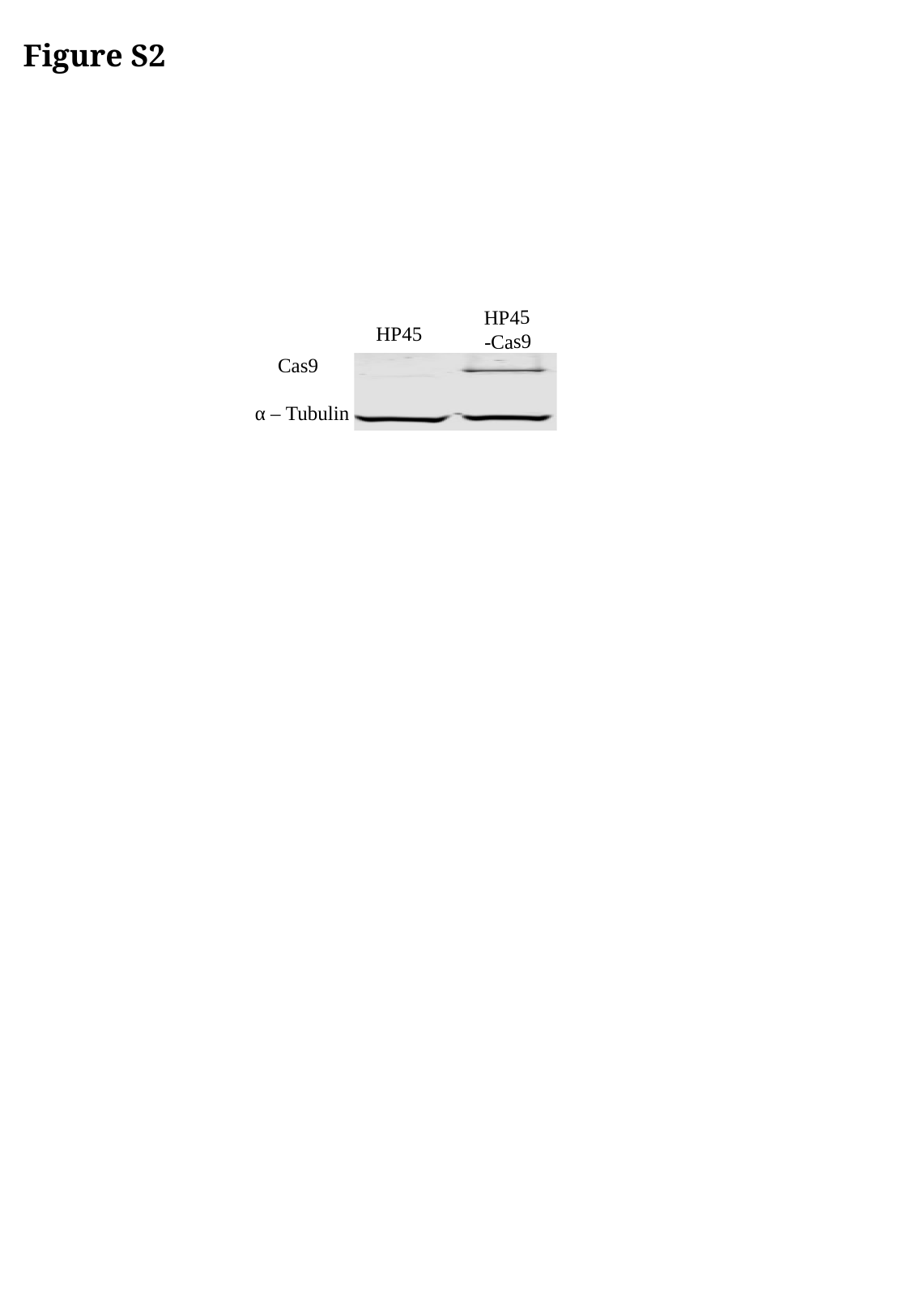

Figure S2
HP45
-Cas9
HP45
Cas9
α – Tubulin

Supplement: Supplementary file 1 [file ijms-23-11263-s001.zip › Supplementary Figures-IJMS.pptx]
